# Online health information-seeking behaviors and skills of Chinese college students

**DOI:** 10.1186/s12889-021-10801-0

**Published:** 2021-04-15

**Authors:** Dangui Zhang, Weixin Zhan, Chunwen Zheng, Jinsheng Zhang, Anqi Huang, Shuan Hu, William Ba-Thein

**Affiliations:** 1 Research Center of Translational Medicine, Second Affiliated Hospital of Shantou University Medical College, Shantou, PR China; 2 Undergraduate Research Training Program (UGRTP), Shantou University Medical College, Shantou, PR China; 3 Clinical Research Unit, Shantou University Medical College, Xinling Road 22, Shantou, 515041 PR China; 4 Department of Microbiology and Immunology, Shantou University Medical College, Xinling Road 22, Shantou, 515041 PR China

**Keywords:** Chinese college students, Online health information, Online behavior, Health literacy, Health risk

## Abstract

**Background:**

Seeking online health information (OHI) has become a common practice globally. The information seekers could face health risks if they are not proficient in OHI literacy. The OHI-seeking behaviors and skills of Chinese college students, the largest proportion of college students in the world, are understudied. This study was aimed to describe OHI-seeking behaviors and skills of college students in Guangdong, China.

**Methods:**

College students in the Guangdong province with OHI-seeking experience were invited via WeChat, QQ, and Sina Weibo using QR code posters and flyers for participation in this online anonymized questionnaire-based study. Data on demographics, OHI literacy, information resources, search approaches, and behaviors were collected. The relationship between perceived OHI literacy and high-risk behaviors was investigated by bivariate logistic regression analysis.

**Results:**

Respondents were 1203 college students with a mean age of 20.6 years, females (60.2%), and undergraduates (97.2%). They sought health information via websites (20.3%), WeChat (2.6%), or both (77.1%). Baidu was the main search engine, and baike.baidu.com (80.3%), Zhihu.com (48.4%), and Zhidao.baidu.com (35.8%) were top three among 20 searched websites for information about self-care (80.7%), general health (79.5%), disease prevention (77.7%), self-medication (61.2%), family treatment (40.9%), drugs (37.7%), western medications (26.6%), hospitals (22.7%), physicians (21.4%), and Traditional Chinese Medicine (15.6%). Despite most respondents (78%) lacked confidence in the evidence quality and satisfaction with the results, only 32.4% further consulted doctors. Many (> 50%) would recommend the retrieved information to others. About 20% experienced hacking/Internet fraud. Cronbach’s alpha for the internal consistency of OHI literacy was 0.786. Bivariate logistic regression analysis showed that students who believed they can judge the evidence level of OHI were more likely to self-diagnose (OR = 2.2, 95%CI, 1.6–3.1) and look for drug usage (OR = 3.1, 95%CI, 1.9–5.0).

**Conclusions:**

This study reveals Chinese college students’ heavy reliance on OHI to manage their own and others’ health without sufficient knowledge/skills to identify misinformation and disinformation. The apparent risky information-seeking behaviors of Chinese college students warrant the provision of regulated, accurate, and actionable health information; assurance of cybersecurity; and health information literacy promotion in colleges by concerned authorities.

**Supplementary Information:**

The online version contains supplementary material available at 10.1186/s12889-021-10801-0.

## Background

The Internet hosts a tremendous amount and variety of health-related information that can be accessed at convenience, anonymity, and relatively low cost. Many health-related websites provide consumer-oriented health information and additional features like a forum (or message board), support groups, and recommended links. Information seekers, therefore, can obtain health information and explore other services as well depending on their motivation of going online, with the resultant outcome as self-efficacy (the extent or strength of one’s belief in one’s own ability to complete tasks and reach goals) in decision making or seeking further professional help [1].

Seeking online health information (OHI) has thus become a common practice globally. The Pew Internet and American Life project in 2013 reported that 59% of American adults used the Internet to access health information [2, 3]. One Eurobarometer survey about European citizens’ digital health information in 2014 showed that 60% of Europeans, mostly aged 16 to 34 years old, searched online for health information [4]. According to the China Internet Network Information Center (CNNIC), there are 829 million Internet users as of December 2018 in China with mobile internet usage accounting for 98.6% [1], but their Internet usage for health information is unknown.

The impact of OHI search is determined by the quality of information and the characteristics of information seekers. While there are known benefits of OHI, such as abundant and easy access to health information, anonymity and privacy in searching sensitive health issues, interactivity with health professionals and peers, and social support [5, 6], the overall quality of consumer-oriented health information on the Internet is reportedly low [7]. Information seekers with poor health information literacy are at health risks of making bad health decisions from misinformation (incorrect information) and disinformation (deliberately disseminated misinformation) because health information literacy as defined by the Medical Library Association is “the set of abilities needed to: recognize a health information need; identify likely information sources and use them to retrieve relevant information; assess the quality of the information and its applicability to a specific situation; and analyze, understand, and use the information to make good health decisions” [8]. Many guidelines on evaluating OHI developed by governmental organizations and academic institutions are available for English-speaking information seekers. However, there are no reliable mechanisms or guidelines for non-English-speaking information seekers to verify the evidence quality of translated or adapted health information.

The Chinese government published the guidelines for health information generation and dissemination in 2015, which highlights the requirements in providing health information, including sources, authors, date of update, target population, application, and references/evidence but with no legal binding on the quality of the information provided [9]. These guidelines are more applicable to website management and less helpful for the public to verify the quality of evidence on Chinese websites.

College students are the population in need of proficient health information literacy skills, as they are in the critical developmental stage to make lifelong healthcare decisions [10]. However, their deficient health information literacy skills have been reported in previous studies [11, 12]. Despite that Chinese college students account for nearly 10% of 829 million Chinese netizens in 2018 [1], sharing the largest proportion of college students in the world [13], their OHI-seeking behaviors and skills are understudied. Our study objective was to describe the behaviors and skills in health information seeking via the Internet and mobile applications among college/university students in Guangdong province, China.

## Methods

### Study design and ethics

This study was a cross-sectional self-administered anonymized online survey. It was approved by the Ethics Committee of Shantou University Medical College (SUMC-2017-34) and reported following the reporting guideline CHERRIES (Checklist for Reporting Results of Internet E-Surveys) [14]. Students from colleges and universities located in Guangdong province, China were invited for voluntary participation with informed consent in the study.

### Questionnaire design and administration

The self-developed survey instrument was designed based on the related literature on the Centers for Disease Control and Prevention (US CDC) websites [15] and informal interviews with the students of Shantou University. We used the qualitative data on the interviewees’ real-life experiences of searching OHI as the template for our survey questionnaire. A total of 20 questions in three pages assessed demographic information, health information literacy, online information-seeking behaviors, and the impact of online search. The content of the survey instrument was standardized and validated by two experts before pilot testing with a group of volunteers (*n* = 20) for usability and technical functionality. Posters and flyers with survey QR code were handed out in 14 universities in the Guangdong province and also posted on popular Chinese social media applications such as WeChat, QQ, and Sina Weibo for voluntary participation in our online survey on a Chinese survey hosting site Sojump (www.wjx.cn) during Apr-May 2018 (see Supplementary File 1 for the English version of the survey). Small monetary incentives were used to encourage participation. Only one IP address per submission was allowed to prevent multiple submissions. The inclusion criteria of participants were 1) students of the colleges/universities in Guangdong province and 2) students with OHI-seeking experience. The participants without OHI-seeking experience (*n* = 167) were identified with a screening question and excluded from the study.

### Data analysis

Only completed questionnaires were analyzed using SPSS version 19 (SPSS Inc., Chicago, IL, USA). 5-item Likert scale (strongly agree, agree, not sure, disagree, strongly disagree) in the survey was converted into a 3-item scale (agree, not sure, disagree) for analysis. The internal consistency (reliability) of the OHI literacy questions analyzed by Cronbach’s alpha was 0.786. Normally distributed continuous variable such as age was analyzed by one-way ANOVA and shown as mean ± SD; categorical variables including sex, academic degree, hometown, perceived online health literacy, behaviors of and experience in seeking OHI, and self-reported beneficial effects of OHI, were analyzed by Chi-square test and shown as n (%). Bivariate logistic regression was performed to analyze the relationship between perceived OHI literacy and high-risk behaviors (self-diagnosis and drug usage). All statistical tests were two-tailed, and *P*-value < 0.05 was considered statistically significant.

## Results

### Characteristics of respondents (Table 1)

During the two-month survey period, there were 1370 visits to the questionnaire website and 1203 (87.8%) were eligible for the study. The mean age of respondents was 20.6 years. The majority of respondents were females (60.2%, 724/1203), undergraduates (97.2%, 1169/1203), and the natives of Guangdong province (84.8%, 1020/1203).

**Table 1:** Characteristics of Chinese college students and online health information-seeking approaches

		Total	Seeking via		
		*N* = 1203	Websites (*n* = 244)	WeChat (*n* = 31)	Both (*n* = 928)
Age (year) ^#^		20.6 ± 3.3	20.3 ± 2.5	18.4 ± 4.2	20.7 ± 3.5
Sex	*Male*	479 (39.8)	103 (42.2)	17 (54.8)	359 (38.7)
	*Female*	724 (60.2)	141 (57.8)	14 (45.2)	569 (61.3)
Degree	*Undergraduate*	1169 (97.2)	235 (96.3)	30 (96.8)	90 (97.4)
	*Postgraduate*	34 (2.8)	9 (3.7)	1 (3.2)	24 (2.6)
Hometown ^&^	*Guangdong*	1020 (84.8)	199 (81.6)	21 (67.7)	800 (86.2)
	*Non-Guangdong*	183 (15.2)	45 (18.4)	10 (32.3)	128 (13.8)

Age shown as mean ± SD was analyzed by one-way ANOVA; sex, education, and hometown shown as n (%) was analyzed by Chi-square test; ^#^ (*P* < 0.001, within groups; *P* = 0.02, Website Vs WeChat; *P* = 0.004, WeChat Vs both); ^&^ *P* = 0.01 (between/within groups)

### Search engines and social media

The respondents reportedly searched health information via websites (20.3%, 244/1203), WeChat (2.6%, 31/1203), or both (77.1%, 928/1203). Baidu was the most used search engine, and among the 20 searched websites, baike.baidu.com (80.3%, 966/1203), Zhihu.com (48.4%, 582/1203), and Zhidao.baidu.com (35.8%, 431/1203) were the top three, followed by dxy.cn (31.0%, 373/1203), wikipedia.org (23.8%, 286/1203), muzhi.baidu.com (17.7%, 213/1203), wanfangdata.com (15.2%, 183/1203), xywy.com (12.7%, 153/1203), guokr.com (12.4%, 149/1203), tieba.baidu.com (10.8%, 130/1203), 39.net (8.7%, 105/1203), health.sohu.com (5.3%, 64/1203), iask.sina.com (2.8%, 34/1203), pubmed.ncbi.nlm.nih.gov (1.4%, 17/1203), medlineplus.gov (0.3%, 4/1203), haodf.com (0.2%, 3/1203), cdc.gov (0.2%, 2/1203), uptodate.cn (0.2%, 2/1203), 120ask.com (0.1%, 1/1203), and medscape.com (0.1%, 1/1203). Of note, many of these websites are related to health, and wikipedia.org was the most visited English website.

### Perceived OHI literacy

**Fig. 1:**
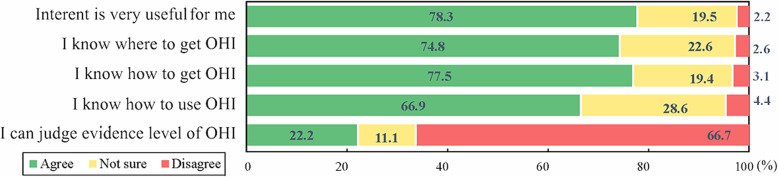
Perceived OHI (online health information) literacy (*n* = 1203)

As shown in Fig. 1, the majority (78.3%, 942/1203) agreed that the Internet was very useful for them and that they had information-seeking skills, including where (74.8%, 900/1203) and how (77.5%, 932/1203) to get the information they want, and how to use the Internet to answer their questions (66.9%, 805/1203). However, only 22.2% (267/1203) of them were confident about the quality of evidence on the Internet.

### OHI-seeking behaviors

#### Motivation and reasons for seeking OHI (Table 2)

More than 90% of participants searched OHI for their families, friends, and/or themselves. The top five reasons for their search were for self-care, general health, disease prevention, self-medication, and treating family members, followed by information about drugs, western medications, hospitals, physicians, and Traditional Chinese Medicines. In comparison, males paid more attention to general health (82.5%, 395/479 vs. 77.5%, 561/724; *P* = 0.04) while females focused more on self-care (85.1% 616/724 vs. 74.1%, 355/479; *P* < 0.001) and self-medication (63.7%, 461/724 vs. 57.4%, 275/479; *P* = 0.03).

**Table 2:** Behaviors and experience of college students in seeking online health information

		Total	Sex			Degree Program		
		(*N* = 1203)	Male (*n* = 479)	Female (*n* = 724)	***P***	UG (*n* = 1169)	PG (*n* = 34)	***P***
For whom	Self	88 (7.3)	41 (46.6)	47 (53.4)	0.49	85 (96.6)	3 (3.4)	0.94
	Families/friends	4 (0.3)	1 (25.0)	3 (75.0)		4 (100)	0 (0)	
	Self + Families/friends	1105 (91.9)	434 (39.3)	671 (60.7)		1074 (97.2)	31 (2.8)	
	Others	6 (0.5)	3 (50.0)	3 (50.0)		6 (100)	0 (0)	
For what	Self-care	971 (80.7)	355 (36.6)	616 (63.4)	< 0.001	945 (97.3)	26 (2.7)	0.51
	General health information	956 (79.5)	395 (41.3)	561 (58.7)	0.04	931 (97.4)	25 (2.6)	0.39
	Disease prevention	935 (77.7)	370 (39.6)	565 (60.4)	0.78	910 (97.3)	25 (2.7)	0.53
	Self-medication	736 (61.2)	275 (37.4)	461 (62.6)	0.03	714 (97.0)	22 (3.0)	0.72
	Treating families	492 (40.9)	183 (37.2)	309 (62.8)	0.13	481 (97.8)	11 (2.2)	0.38
	Drug information	453 (37.7)	174 (38.4)	279 (61.6)	0.47	437 (96.5)	16 (3.5)	0.28
	Western medication	320 (26.6)	128 (40.0)	192 (60.0)	0.95	308 (96.3)	12 (3.8)	0.24
	Hospital information	273 (22.7)	105 (38.5)	168 (61.5)	0.62	261 (95.6)	12 (4.4)	0.09
	Physician information	257 (21.4)	103 (40.1)	154 (59.9)	0.94	247 (96.1)	10 (3.9)	0.29
	TCM treatment	188 (15.6)	66 (35.1)	122 (64.9)	0.17	184 (97.9)	4 (2.1)	0.64
	Others	165 (13.7)	68 (41.2)	97 (58.8)	0.73	160 (97.0)	5 (3.0)	0.80
Decision making after discussion with	Self	813 (67.6)	326 (40.1)	487 (59.9)	0.80	789 (97.0)	24 (3.0)	0.85
	Families	416 (34.6)	153 (36.8)	263 (63.2)	0.12	406 (97.6)	10 (2.4)	0.59
	Doctors	390 (32.4)	172 (44.1)	218 (55.9)	0.04	376 (96.4)	14 (3.6)	0.27
	Friends	387 (32.2)	140 (36.2)	247 (63.8)	0.08	373 (96.4)	14 (3.6)	0.27
Sharing the retrieved information with friends		645 (53.6)	271 (42.0)	374 (58.0)	0.09	629 (97.5)	16 (2.5)	0.60
Recommending the retrieved information to others		679 (56.4)	289 (42.6)	390 (57.4)	0.02	660 (97.2)	19 (2.8)	0.94
Safety concerns	No	334 (27.8)	149 (44.6)	185 (55.4)	0.02	322 (96.4)	12 (3.6)	0.69
	Hacking attack	75 (6.2)	31 (41.3)	44 (58.7)		74 (98.7)	1 (1.3)	
	Internet fraud	511 (42.5)	207 (40.5)	304 (59.5)		498 (97.5)	13 (2.5)	
	Both	283 (23.5)	92 (32.5)	191 (67.5)		275 (97.1)	8 (2.9)	
Victim experience	Hacking attack	53 (4.4)	33 (62.3)	20 (37.7)	0.001	53 (100)	0 (0)	0.40
	Internet fraud	189 (15.7)	111 (58.7)	78 (41.3)	< 0.001	183 (96.8)	6 (3.2)	0.81
Acceptability of retrieved information	Yes	635 (52.8)	263 (41.4)	372 (58.6)	0.09	616 (97.0)	19 (3.0)	0.02
	Not sure	461 (38.3)	167 (36.2)	294 (63.8)		453 (98.3)	8 (1.7)	
	No	107 (8.9)	49 (45.8)	58 (54.2)		100 (93.5)	7 (6.5)	

Data shown as n (%) and proportions were analyzed by Chi-square test); *UD* Undergraduates, *PD* Postgraduates, *TCM* Traditional Chinese medicine

#### Health decision making and information management (Table 2)

The majority (ca. 70%) of respondents made decisions by themselves or with families and friends based on the information they retrieved without further consultation with doctors. The proportion of females was significantly higher than that of males among those who consulted doctors (55.9% vs. 44.1%, *P* = 0.04).

Although the majority of the respondents (77.9%, 937/1203) were not satisfied with their sought results (data not shown), many of them considered the retrieved online information acceptable (52.8%, 635/1203), would share with others (53.6%, 645/1203), and would recommend others (56.4%, 679/1203). Females were more likely to recommend their searched information to others than males (57.4% vs. 42.6%, *P* = 0.02).

### Impact of seeking OHI

#### Benefits (Fig. 2)

**Fig. 2:**
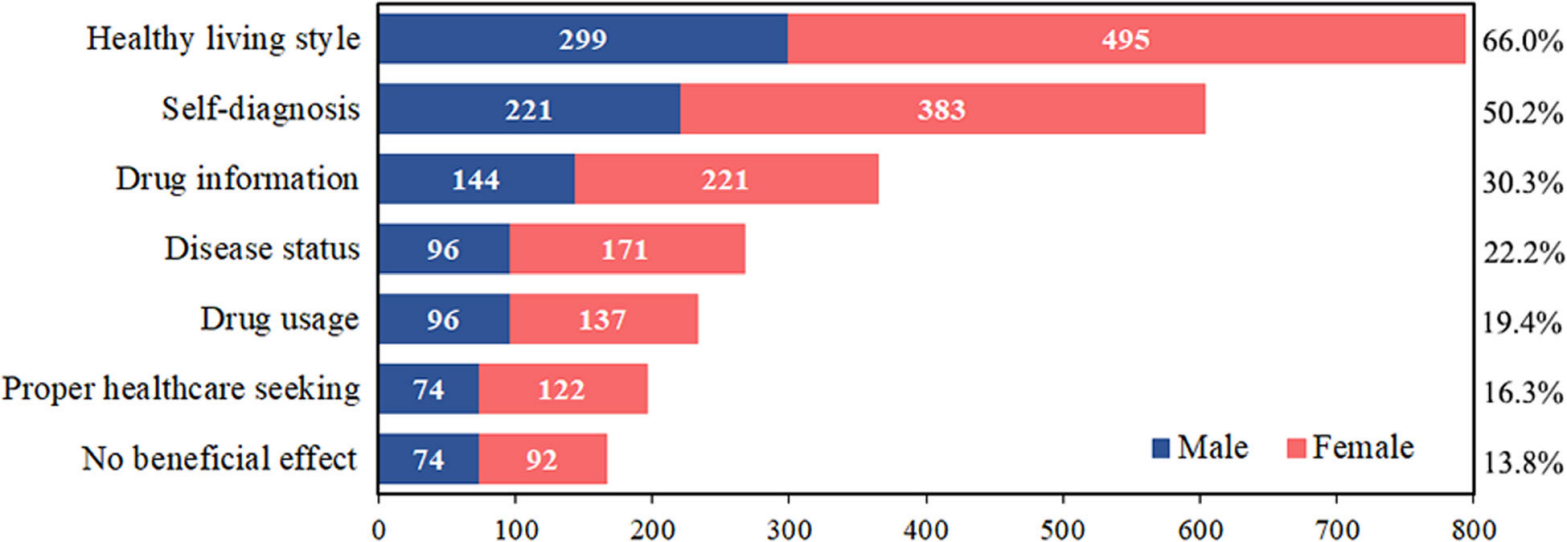
Self-reported beneficial effects of OHI (online health information) (*n* = 1203)

Figure 2 shows self-reported benefits from online information. The benefits were related to healthy living style (66.0%, 794/1203), followed by self-diagnosis (50.2%, 604/1203), drug information (30.3%, 365/1203), diseases status (22.2%, 267/1203), drug usage (19.4%, 233/1203), and proper healthcare-seeking (16.3%, 196/1203). Only a minority of respondents (13.8%, 166/1203) claimed no beneficial effect of the retrieved health information on themselves or their families.

#### Risks (Table 2 and Fig. 3)

**Fig. 3:**
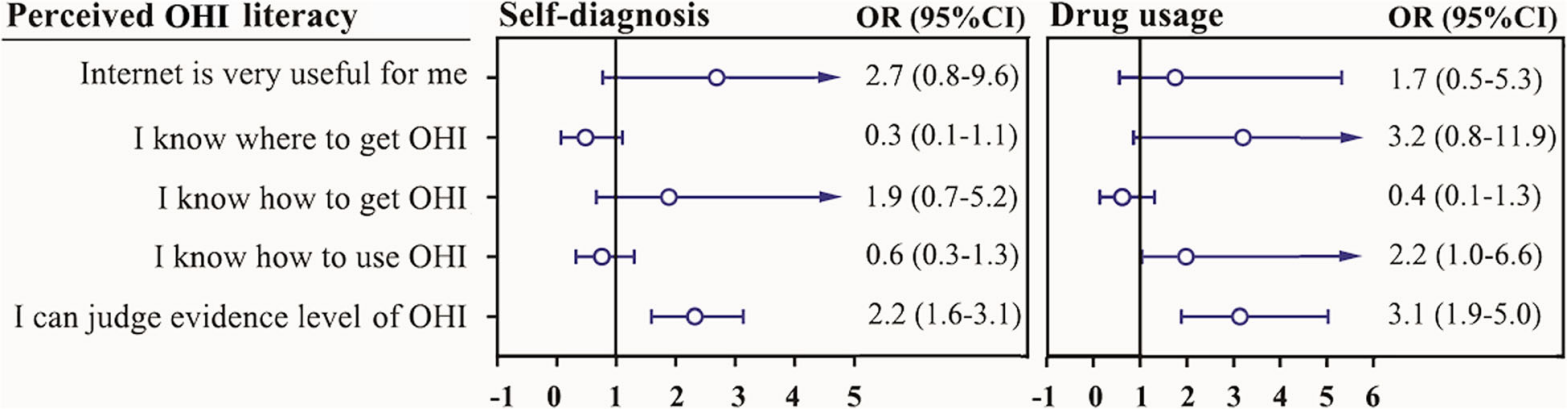
Bivariate logistic regression of risk behaviors (self-diagnosis and drug usage) among college students and their perceived OHI (online health information) literacy (agree vs. disagree as a reference)

As shown in Table 2, although nearly 30% of respondents were unconcerned about hacking attack or Internet fraud when browsing websites, up to 20.1% of them declared that they fell victim to hacking attacks (4.4%, 53/1203) or Internet fraud (15.7%, 189/1203), with more males than females being involved (hacking, *P* = 0.001; fraud, *P* < 0.001; respectively). Figure 3 (bivariate logistic regression analysis) shows that students who believed they could judge the evidence level of OHI were more likely to self-diagnose (OR = 2.2, 95% CI, 1.6–3.1) and look for drug usage (OR = 3.1, 95% CI, 1.9–5.0).

## Discussion

This paper describes Internet use for health information and the online behaviors and skills among college students in Guangdong—the most populous province of China. Our study revealed the most popular search engines, websites, and social media among Chinese college students; their perceived OHI literacy; their OHI seeking behaviors including their motivation and reasons for seeking OHI, health decision making and information management; and the benefits and risks from seeking OHI.

There are some studies recently published on OHI in China. One focus group qualitative study with 27 college students in a mid-sized university in Shanxi Province [16] reported the sources of health information and the search behaviors of Chinese undergraduate students. Another study with Chinese college students focused on online mental health information seeking [17] and showed that the participants were sensitive to the quality of search platform service, rather than the information quality. Although the findings of these studies have partially exposed the OHI-seeking behaviors of college students, with their sample size and study topic limitations, they could not reflect the real situation in a larger population of Chinese college/university students like in this study.

### OHI-seeking behaviors

College students in English-speaking countries seek health information via two common platforms —via multiple search engines (e.g. Google, Bing, or Yahoo) or social media (e.g. Facebook or Twitter) [18]. Chinese college students in this study, however, mainly used Baidu search engine and WeChat social media application exclusively in Chinese, which illustrates that regardless of differences in language and search platforms, search strategies among university students are similar [16, 19, 20]. As reported in previous studies [11, 12], among Chinese college students, females were more likely to go online for health information.

Reasons for Chinese college students seeking OHI are varied. More than 60% of the respondents went online for self-diagnosis or self-medication. Self-diagnosis is not encouraged as it causes undue anxiety in the patients especially if they are cyberchondriacs (individuals who compulsively search for health information, triggering undue health anxiety) [21], interferes with doctor-patient relationships [22], and is potentially subject to financial exploitation by e-health organizations and pharmaceutical companies [21, 22]. Self-diagnosing prodromal or early symptoms, however, may be helpful for those who would later consult their physicians to receive proper diagnosis and treatment. But many of our respondents intended to self-medicate themselves without a physician’s assistance, which is consistent with our previous findings that self-medication is prevalent among Chinese university students [23, 24].

Self-medication has become a global health problem for its risks, such as treatment of misdiagnosed medical problems with over-the-counter medicines, adverse drug effects, drug interactions, dosage or treatment duration errors, and drug addiction or abuse [24, 25].

More than 90% of respondents reported that they turned to online sources of health information for themselves as well as for others. This rate of proxy information seekers is extremely high compared to 50% in American health information seekers [3]. One likely reason is that Chinese college students are more acquainted with technology than are their families and relatives.

The most concerning issue in China is that most health information hosted on the Chinese websites or social media is translated and doctored versions of primary information in English with translational errors, personal opinions, and commercial interest links to drug companies, health-related vendors and suppliers, hospitals, and physicians, rarely providing the primary source of information (unpublished data from our investigation on the evidence quality of two most sought health issues—H1N1 influenza prevention and hypertension treatment—in the 20 websites visited by the respondents); therefore, it is impossible, even for experienced medical professionals, to verify the quality (accuracy, completeness, reliability, currency) and authenticity of the information.

Without having sufficient knowledge and skills to judge the quality of OHI, which was reflected in their self-assessment, more than half of the respondents still would not only accept the information they retrieved but also share it with or recommend it to others. Since information seekers’ judgment on the OHI quality is reportedly highly subjective and influenced by personal beliefs [7], college students involved in various OHI could become providers of medical misinformation [26] or even online opinion leaders. These altogether suggest that the impact of any misinformation or disinformation shared or exchanged online can be exponential and thus significant for public health.

Another concern is cybersafety. Even though the Internet is a known place of hackers and fraudsters [27], nearly 30% of the respondents were not cautious about hacking attacks or Internet frauds when browsing websites, and 20% of the respondents having fallen victim to cyber frauds, indicating their poor attitude towards cyber safety and lack of cybersecurity literacy (i.e., knowledge, skills, and attitudes towards recognizing cybersecurity risk).

### OHI literacy competency

Health information literacy is conceived as a combination of health literacy and information literacy [8]. The US Institute of Medicine (IOM) defines health literacy as “the degree to which individuals have the capacity to obtain, process, and understand basic health information and services needed to make appropriate health decisions”. The health literacy rate of Chinese citizens in a nationwide study was only 14% in 2017 [28], which was conducted after the Ministry of Public Health of China had launched Health Literacy Promotion Initiatives with “Health Literacy 66”, i.e., 66 health literacy goals for Chinese citizens, in 2016 [29].

With such considerably low China’s health literacy rate, it is not surprising to observe inadequate health information literacy competency in this study, which is reflected by the fact that the majority of students were neither confident in the quality and trustworthiness of the online information nor satisfied with the searched results, and yet they would share the information with or recommend it to others. Those with poor OHI are at risk of succumbing to unsubstantiated misinformation and disinformation on unregulated websites. Even for health information literacy-competent information seekers, infodemic—an overabundance of information including misinformation—on the Internet could be overwhelming, confusing, and misleading.

### Benefits and risks

Unquestionably, there are benefits and risks of OHI [6]. While a mere minority of respondents in this study found the Internet unhelpful, the sought information appeared to be beneficial for many respondents at least regarding adopting a healthy living style and health-seeking behavior. On the other hand, with their heavy reliance on online information, many respondents were at high risk of health mismanagement, especially self-diagnosis and self-medication among those with perceived competency in OHI literacy (Fig. 3). Chinese female college students had a higher risk as they were more likely to seek OHI for self-diagnosis and self-medication.

One noteworthy aspect of OHI is its impact on the risk perception and health behaviors of information seekers with poor health information literacy. As reported in media outlets across the world, the current pandemic coronavirus disease 2019 (COVID-19) has seen incompliant and irrational public responses towards infection control and prevention measures in various countries. One of the likely reasons could relate to inadequate health literacy across the globe, which is represented by the fact that the health literacy rate of nearly half of all Europeans in 2013 [30], 57% of American adults in 2016 [31], and 86% of mainland Chinese in 2017 was inadequate [28].

### Need for effective interventions

Even with decade-long efforts to address citizen’s health literacy in many countries [29], poor health literacy remains one of the most pressing public health issues globally. Therefore, some countries have taken enhanced actions to improve the situation; for example, the US’s Healthy People 2020 [32] and the eHealth Action Plan 2012–2020 by the European Commission outline approaches to provide accurate, assessable, and actionable health information and promote OHI literacy [4]. Chinese government as well launched Healthy China 2030 [33], but specific actions or guidance for the netizens and authorities concerned are yet to come.

Despite its formidable task, monitoring and regulation of unsubstantiated OHI is an inevitable public health measure for any country. Given that OHI on Chinese websites is not firsthand and also unregulated, offering an official guide on how to find reliable health information by government agencies such as the one sponsored by the US Department of Health and Health Services [34], promoting the Health on the Net Foundation Code of Conduct (HONcode) certification [35], or providing a user-friendly and trustworthy OHI resource, similar to MedlinePlus [36], for the general public and healthcare providers is required in China for its 829 million netizens [1].

### Limitations

This study had some limitations intrinsic to self-reported online surveys and specific to the complex nature of the Chinese Internet and netizen behaviors. Even though popular search engines, websites, and social media such as Google, YouTube, Facebook, Twitter, Instagram, and Snapchat are not available in China, Chinese netizens still can access them using illegal virtual private networks (VPNs). Therefore, our findings may not reflect their true online behaviors. It should also be cautious in interpreting the information-seeking behaviors of college students because only those with experience of searching OHI participated in this study.

## Conclusion

This study reveals Chinese college students’ heavy reliance on OHI to manage their own and others’ health without sufficient knowledge and skills to identify misinformation and disinformation, thereby placing themselves and others at health risk. As the major netizens and potential health information providers and opinion leaders, college students should have adequate health information literacy. Their risky information-seeking behaviors warrant the provision of regulated, accurate, and actionable health information, assurance of cybersecurity, and promoting health information literacy in colleges by the concerned authorities.

## Supplementary Information

**Additional file 1.** Questionnaire: Online Health Information-seeking behaviors.

## Data Availability

The datasets generated and/or analyzed during the current study are not publicly available due to confidentiality as they include sensitive private information but are available from the corresponding author on reasonable request.

## Abbreviations

OHI: Online health information
CNNIC: China Internet Network Information Center
CHERRIES: Checklist for Reporting Results of Internet E-Surveys
IOM: US Institute of Medicine
VPNs: Virtual private networks
HONcode: Health on the Net Foundation Code of Conduct
