# Supplementary material for: Online health information-seeking behaviors and skills of Chinese college students
Source: BMC Public Health. 2021 Apr 15;21:736. doi: 10.1186/s12889-021-10801-0 (PMC8047546; doi:10.1186/s12889-021-10801-0)
Supplement: Supplementary file 1 — Additional file 1. Questionnaire: Online Health Information-seeking behaviors. [file 12889_2021_10801_MOESM1_ESM.docx]

**Questionnaire: Online Health Information-seeking behaviors**

- **Screening Questions**

Do you search health-related information online? YES_____, NO ____

Do you search health-related information by social network app? YES_____, NO _____

If **NO, you are not eligible for participation in this study. Thanks for your interest.**

- **Participant`s background**

1. Your age: ____ years
2. Your sex: male _____ female _____
3. You are living in ________________________________ city, __________________________ province
4. Your education:
   1. No education _____
   2. Highest level of your education:
      1. Primary school _____
      2. Middle school _____
      3. High school _____
      4. Vocational school _____
      5. College _____
5. Your occupation
   1. Current: __________________________________________________________________________________
   2. Past: __________________________________________________________________________________

- **Knowledge and behaviors about online health information seeking**

1. Perceived Internet literacy (5= strongly agree, 4= agree, 3= undecided, 2= disagree, 1= strongly disagree)
   1. The Internet is very useful for me. _____
   2. I know **where to find** helpful resources for information I want on the Internet. _____
   3. I know **how to find** helpful resources for information I want on the Internet. _____
   4. I know **how to use** the Internet to answer my questions. _____
   5. I can distinguish high-quality resources from low-quality resources on the Internet. _____
2. Your reasons for online health information seeking were: *(multiple choice)
   1. for myself_____, for others _____, both _____
   2. for prevention of diseases _____
   3. for better health _____
   4. for receiving treatment _____
3. The information you sought was for: *(multiple choice)
   1. Home remedies _____; specify ________________________________________________________________
   2. Self-medication _____; specify ________________________________________________________________
   3. Checking the prescribed medication _____; specify _______________________________________________
   4. Finding healthcare providers _____
   5. Finding hospitals _____
   6. Others _____; specify ________________________________________________________________________
4. How did you get health information?
   1. From mobile app: WeChat_____, QQ_____, Mum Group_____, Rain Dr_____, DXY.com_____, Keep _____, Zhihu.com_____, Others _____
   2. From the websites linked to the above social media sites _____
   3. From the websites assessed directly using personal computer _____
5. Please list the websites (sources of health information) you visited in last 6 months: ______________________________________________________________________________________________________________________________________________________________________________________________
6. When you received the online health-related information, you made decision
   1. By yourself ______
   2. After discussing with your family ______
   3. After discussing with your friends ______
   4. After discussing with your physicians ______

- **The impact of online health information seeking**

1. Level of satisfaction with the online health information you received ______

(5= very high, 4= high, 3= moderate, 2= low, 1= not at all)

1. Are you aware of the following risk when searching online?
   1. Hacking YES______, NO ______
   2. Scam YES______, NO ______
2. Have you ever been hacked? YES______, NO ______
3. Have you ever been scammed? YES______, NO ______
4. Does online health information have a positive impact on your health? YES______, NO ______
   1. Give examples: ______________________________________________________________________
5. Does online health information have a positive impact on my family`s health? YES______, NO ______
   1. Give examples: ______________________________________________________________________
6. What is your opinion about online health information? ______________________________________________________________________________________________________________________________________________________________________________________________
7. Will you recommend others to search health information online? YES______, NO ______
8. Will you forward health-related information to your friends? YES______, NO ______
